# Treatment of hypertension in children and adolescents

**DOI:** 10.1007/s00467-007-0573-4

**Published:** 2009-10-01

**Authors:** Marc B. Lande, Joseph T. Flynn

**Affiliations:** 1 Pediatric Nephrology, University of Rochester, Rochester, NY USA; 2 Division of Nephrology, A-7931, Children’s Hospital & Regional Medical Center, 4800 Sand Point Way NE, Seattle, WA 98105 USA

**Keywords:** Children, Hypertension, Treatment, Adolescent, Kidney diseases, Obesity, Athletes, Metabolic syndrome

## Abstract

The treatment of hypertension in children and adolescents has been markedly changed in recent years by several factors, including the publication of new consensus recommendations, the obesity epidemic, and the increased availability of information on efficacy and safety of antihypertensive medications in the young. In this review we present an updated approach to the outpatient management of hypertension in the child or adolescent, utilizing representative cases to illustrate important principles as well as possible controversies.

## Definition, classification and identification of hypertension

In adults, the definition of hypertension is based upon outcomes such as myocardial infarction and stroke; large-scale population studies have demonstrated that the incidence of these events increases in a linear fashion, beginning with blood pressures >120/80 mmHg [1]. Since such outcomes are exceedingly rare in children and adolescents, the definition of elevated blood pressure in the young is a statistical one, derived from databases of blood pressures obtained in healthy children. The most recent classification scheme for blood pressure in children and adolescents is that recommended by the United States National High Blood Pressure Education Program (NHBPEP) Working Group in 2004 (Table 1) [2]. Although this scheme has its flaws (for example, reliance on one blood pressure measurement per subject, obtained with a mercury sphygmomanometer), it has been generally adopted worldwide.

**Table 1:** Classification of elevated blood pressure in children and adolescents ≤17 years of age (adapted from [2])

Classification	Blood pressure value
Normal	<90th percentile^a^
Pre-hypertensive	≥90th and <95th percentiles or ≥120/80 mmHg in adolescents
Hypertensive	≥95th percentile
Stage 1 hypertension	95th to 99th percentile +5 mmHg
Stage 2 hypertension	≥99th percentile +5 mmHg

^a^See tables published in [2] (Fourth Report)

Current consensus recommendations from the NHBPEP state that blood pressure be measured in all children and adolescents ≥3 years old at all medical encounters, as well as in selected children <3 years old at risk for hypertension [2]. Since blood pressure is known to be quite labile in childhood, and since there is a high prevalence of the “white coat” effect in the young, it is recommended that elevated blood pressure measurements be repeated before a child is identified as hypertensive and a diagnostic evaluation is undertaken. The NHBPEP Working Group definition of hypertension is actually based on the child’s having an elevated blood pressure documented on three occasions. The full list of normative blood pressure (BP) values is available in the Fourth Report [2], which can be obtained free on-line at https://doi.org/www.nhlbi.nih.gov/health/prof/heart/hbp/hbp_ped.htm. These recommendations also include guidance on how soon repeat blood pressures should be obtained after it has been confirmed that a child or adolescent has an elevated BP, and when and how soon a diagnostic workup should be initiated (Table 2).

**Table 2:** Recommended frequency for repeat blood pressure measurements in children and adolescents ≤17 years of age (adapted from [2])

BP level^a^	Frequency of BP measurement
Normal	Recheck at next regularly scheduled physical examination
Pre-hypertensive	Recheck in 6 months
Stage 1 hypertension	Recheck in 1–2 weeks or sooner if the patient is symptomatic; if BP is persistently elevated on two additional occasions, evaluate or refer to source of care within 1 month
Stage 2 hypertension	Evaluate or refer to source of care within 1 week or immediately if the patient is symptomatic

^a^Based upon classification scheme in Table 1

One potential flaw of using office blood pressure measurements alone is the possibility of mis-identifying patients as having hypertension when, in fact, their blood pressures are normal. This phenomenon, known as white-coat hypertension, may be seen in up to 60% of children referred for the evaluation of elevated blood pressure [3]. It is important to identify white-coat hypertension, since it does not appear to be associated with the development of hypertensive target-organ damage, meaning that further evaluation can be avoided. The opposite phenomenon, termed masked hypertension, is seen when office blood pressures are normal but the child or adolescent is actually hypertensive. Masked hypertension is especially important to identify in children with underlying renal disease, in whom elevated blood pressures may contribute to progression. Both of these phenomena can be detected by ambulatory blood pressure monitoring [3], a technique that is finding greater application in the evaluation of children with elevated blood pressure. We feel that ambulatory blood pressure monitoring (ABPM) should be used to confirm hypertension in otherwise healthy children and adolescents, and to confirm normotension in those with chronic kidney disease (CKD), as part of the initial evaluation of such patients.

## Guiding principles of hypertension management

After the diagnosis of hypertension has been confirmed and the underlying etiology (if any) identified, an individualized treatment regimen should be initiated. Most authorities recommend that this include non-pharmacologic measures for all patients, with addition of antihypertensive medications in a selected group of children (Table 3). Medications are not recommended for all patients, since there are no long-term pediatric data on their benefits or adverse effects on growth and development. Although recent legislative initiatives in the USA and Europe have produced, and will continue to produce, substantial data on the short-term efficacy and tolerability of antihypertensive agents in children and adolescents [4], it is unlikely that studies of sufficient duration to answer the long-term questions will ever be conducted in the young.

**Table 3:** Indications for antihypertensive medications in hypertensive children and adolescents (adapted from [2])

Indications	
•	Stage 2 hypertension
•	Symptomatic hypertension
•	Secondary hypertension
•	Hypertensive target-organ damage
•	Diabetes (types 1 and 2)
•	Persistent hypertension despite non-pharmacologic measures

Non-pharmacologic approaches to hypertension generally consist of dietary changes, increased physical activity and weight loss in the obese. Although it is difficult to apply each of these measures successfully in practice, evidence supporting their efficacy in children and adolescents exists, so they remain a cornerstone of recent consensus recommendations [2]. Furthermore, studies conducted in adults have demonstrated that successful lifestyle modification may enhance the efficacy of antihypertensive drug therapy [1]. While a detailed review of these approaches is beyond the scope of this article, we will review pertinent aspects of non-pharmacologic management in the case discussions below.

Antihypertensive drug prescribing in children and adolescents should generally begin with the physician’s choosing an agent appropriate to the underlying etiology of the patient’s hypertension. An example of this is the use of diuretics in acute glomerulonephritis or other forms of volume-overload hypertension. Other specific examples of appropriate initial drug choice will be provided in the case discussions. Controversy does remain with respect to choice of initial agent when there is no specific underlying etiology of hypertension identified. In adults, a thiazide diuretic is usually chosen, based upon evidence of the superiority of this class in reducing cardiovascular morbidity and mortality in large-scale trials such as the Antihypertensive and Lipid-Lowering Treatment to Prevent Heart Attack Trial (ALLHAT), in which multiple classes of antihypertensive agents were compared [5]. Given the lack of comparable studies in the young, the choice of initial agent in children with primary hypertension remains up to the individual practitioner. No matter what agent is chosen, it is advisable from several standpoints to prescribe only those agents that have established pediatric indications and/or labeling information [2]. Fortunately, the number of such agents has greatly increased over the past 10 years, and detailed information on the efficacy of specific agents in hypertensive children and adolescents is now available [4]. Recommended doses for drugs used in outpatient treatment of hypertension in children and adolescents can be found in Table 4.

**Table 4:** Recommended doses for selected antihypertensive agents for outpatient management of hypertension in children and adolescents (*b.i.d.* twice daily, *HCTZ* hydrochlorothiazide, *q.d.* once daily, *q.i.d.* four times daily, *t.i.d.* three times daily)

Class	Drug	Starting dose	Interval	Maximum dose^a^
Aldosterone receptor antagonists	Eplerenone	25–50 mg/day	q.d.–b.i.d.	100 mg/day
	Sprionolactone^b^	1 mg/kg per day	q.d.–b.i.d.	3.3 mg/kg per day up to 100 mg/day
Angiotensin-converting enzyme inhibitors	Benazepril^b^	0.2 mg/kg per day up to 10 mg/day	q.d.	0.6 mg/kg per day up to 40 mg q.d.
	Captopril^b^	0.3–0.5 mg/kg per dose	b.i.d.–t.i.d.	6 mg/kg per day up to 450 mg/day
	Enalapril^b^	0.08 mg/kg per day	q.d.	0.6 mg/kg per day up to 40 mg/day
	Fosinopril	0.1 mg/kg per day up to 10 mg/day	q.d.	0.6 mg/kg per day up to 40 mg/day
	Lisinopril^b^	0.07 mg/kg per day up to 5 mg/day	q.d.	0.6 mg/kg per day up to 40 mg/day
	Quinapril	5–10 mg/day	q.d.	80 mg/day
	Ramipril	2.5 mg/day	q.d.	20 mg/day
Angiotensin-receptor blockers	Candesartan	4 mg/day	q.d.	32 mg/day
	Irbesartan	75–150 mg/day	q.d.	300 mg/day
	Losartan^b^	0.75 mg/kg per day up to 50 mg/day	q.d.	1.4 mg/kg per day up to 100 mg/day
	Valsartan	0.25 mg/kg per day up to 80 mg/day	q.d.	4 mg/kg per day up to 320 mg/day
α-and β-adrenergic antagonists	Labetalol^b^	2–3 mg/kg per day	b.i.d.	10–12 mg/kg per day up to 1.2 g/day
	Carvedilol	0.1 mg/kg/dose up to 12.5 mg b.i.d.	b.i.d.	0.5 mg/kg per dose up to 25 mg b.i.d.
β-adrenergic antagonists	Atenolol^b^	0.5–1 mg/kg per day	q.d.–b.i.d.	2 mg/kg per day up to 100 mg/day
	Bisoprolol/HCTZ	0.04 mg/kg/day up to 2.5/6.25 mg/day	q.d.	10/6.25 mg/day
	Metoprolol	0.5–1.0 mg/kg per day up to 50 mg/day	q.d. (extended-release)	2 mg/kg per day up to 200 mg/day
	Propranolol	1 mg/kg per day	b.i.d.–t.i.d.	16 mg/kg per day up to 640 mg/day
Calcium channel blockers	Amlodipine^b^	0.06 mg/kg per day up to 5 mg/day	q.d.	0.6 mg/kg per day up to 10 mg/day
	Felodipine	2.5 mg/day	q.d.	10 mg/day
	Isradipine^b^	0.05–0.15 mg/kg per dose	t.i.d.–q.i.d.	0.8 mg/kg per day up to 20 mg/day
	Extended-release nifedipine	0.25–0.5 mg/kg per day	q.d.–b.i.d.	3 mg/kg per day up to 120 mg/day
Central α-agonists	Clonidine^b^	5–10 μg/kg per day	b.i.d.–t.i.d.	25 μg/kg per day up to 0.9 mg/day
	Methyldopa^b^	5 mg/kg per day	b.i.d.–q.i.d.	40 mg/kg per day up to 3 g/day
Diuretics	Amiloride	5–10 mg/day	q.d.	20 mg/day
	Chlorothiazide	10 mg/kg per day	b.i.d.	20 mg/kg per day up to 1.0 g/day
	Chlorthalidone	0.3 mg/kg per day	q.d.	2 mg/kg per day up to 50 mg/day
	Furosemide	0.5–2.0 mg/kg per dose	q.d.–b.i.d.	6 mg/kg/day
	HCTZ	0.5–1 mg/kg per day	q.d.	3 mg/kg per day up to 50 mg/day
	Triamterene	1–2 mg/kg per day	b.i.d.	3–4 mg/kg per day up to 300 mg/day
Peripheral α-antagonists	Doxazosin	1 mg/day	q.d.	4 mg/day
	Prazosin	0.05–0.1 mg/kg per day	t.i.d.	0.5 mg/kg per day
	Terazosin	1 mg/day	q.d.	20 mg/day
Vasodilators	Hydralazine	0.25 mg/kg per dose	t.i.d.–q.i.d.	7.5 mg/kg per day up to 200 mg/day
	Minoxidil	0.1–0.2 mg/kg per day	b.i.d.–t.i.d.	1 mg/kg per day up to 50 mg/day

^a^The maximum recommended adult dose should never be exceeded

^b^Preparation of a stable extemporaneous suspension is possible for these agents

**Fig. 1:**
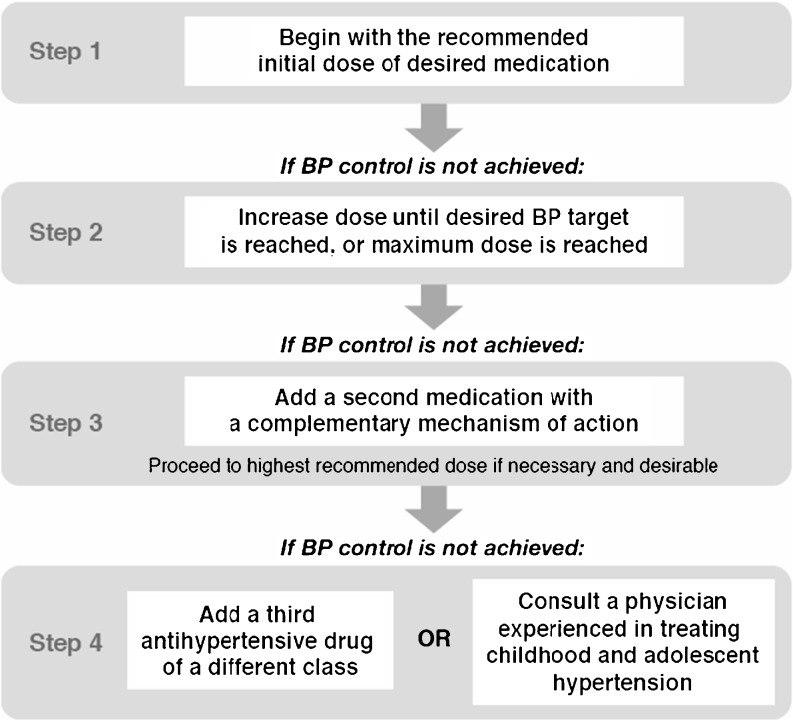
Stepped-care approach to antihypertensive therapy

Once an initial antihypertensive agent has been chosen, a stepped-care approach (Fig. 1) [4] should be followed. Stepped-care allows for the individualization of therapy according to the needs of the patient and also facilitates detection of adverse effects as drug doses are increased or new agents added. It has been endorsed by the last three pediatric working groups of the NHBPEP [2] as an appropriate approach to the use of antihypertensive drugs in children and adolescents. After initiation of drug therapy, follow-up visits should be scheduled frequently (every 2–4 weeks) until blood pressure control has been achieved, and then less frequently (every 3–4 months) thereafter. Home blood pressure monitoring and assessment for medication side-effects are important components of treatment and should be reviewed at each follow-up visit. Periodic reassessment for hypertensive target-organ damage and laboratory monitoring, as appropriate, should also be incorporated into each child’s treatment plan.

## Case presentations

In the remainder of the article we have taken a case-based approach to the discussion of hypertension management. Instead of a straightforward discussion of lifestyle changes, medication choice, etc., we instead present three hypothetical patients that are similar to many children we have evaluated and treated for hypertension. We feel that this format lends itself to a more realistic discussion of key management issues that overlaps with other aspects such as diagnosis and prognosis.

### The hypertensive athlete

*S. is a 14-year-old soccer player referred for evaluation of elevated blood pressure detected at a pre-sports participation screening at her school. Blood pressures obtained at the screening ranged from 137–149/75–80 mmHg. She is at the 50th percentile for height and weight and has no other chronic health problems or abnormal physical examination findings. Because of the elevated BPs, her soccer coach will not allow her to participate on the team. She and her family have asked you to write a letter allowing her to play without restrictions.*

Over 30 million children and adolescents in the USA participate in organized team sports [6]. When the young athlete is found to be hypertensive, several issues arise regarding the clinical evaluation, sports eligibility, and best therapy.

The clinical history of the hypertensive athlete should include questions about the use of performance-enhancing substances, such as anabolic steroids, herbs, supplements, caffeine-containing energy drinks, and stimulants, as many of these can elevate blood pressure. The prevalence of anabolic steroid use among high-school athletes is as high as 3–7%, and approximately 10% of anabolic steroid users are teenagers [6, 7]. Findings on physical examination that should increase the suspicion for steroid use include increased muscle mass, acne, and striae; gynecomastia, and testicular atrophy in males; and facial hair, deep voice, baldness, and breast atrophy in females [6]. Athletes should be questioned about the use of non-steroidal anti-inflammatory drugs, and females about oral contraceptives, as both can raise blood pressure [8, 9]. Twenty-four hour ABPM should be strongly considered in order to exclude white-coat hypertension in athletes with elevated office blood pressure. The evaluation of all athletes with confirmed hypertension should include analyses for levels of blood urea nitrogen (BUN), creatinine, and electrolytes; urinalysis, renal ultrasound, fasting lipid profile, and echocardiogram [2]. Fit athletes with no identifiable risk factors for hypertension on current history, past medical history, or family history should have further investigation for renovascular hypertension and other secondary causes of hypertension, according to the recommendations of the NHBPEP Working Group [2].

The current role of exercise stress testing in the evaluation of the hypertensive youth is not well defined. While exercise testing can be used to test the functional capacity for athletic activities, the American College of Sports Medicine does not recommend routine exercise testing of the hypertensive athlete [10–12]. Furthermore, neither adult nor pediatric expert panels identify exercise stress testing as part of the routine evaluation of individuals in whom hypertension is the only cardiovascular abnormality [2, 10, 13].

Providers sometimes restrict hypertensive youth from athletics for fear that elevated BP values reached during exercise will lead to morbid events such as stroke or myocardial ischemia. The concern stems, in part, from the experience with vigorous exercise in hypertensive adults [11]. Blood pressure response to dynamic exercise differs from the response to static exercise. Normally, dynamic exercise raises systolic BP due to increased cardiac output. Conversely, diastolic BP remains unchanged or decreases due to decreased peripheral vascular resistance. During static exercise, both systolic and diastolic pressures rise and often to a higher level [13]. In comparison to normotensive subjects, hypertensive children and adults can have an exaggerated increase in systolic BP and increased diastolic pressure during dynamic exercise testing [12, 14]. In adults with atherosclerotic disease, the exaggerated blood pressure response to exercise can be associated with mild systolic dysfunction and is predictive of incident cardiovascular events [15, 16]. Furthermore, sudden death due to vigorous exertion has been reported in hypertensive adults over 35 years old who have atherosclerotic coronary artery disease [17].

However, the experience in hypertensive youth differs from that of adults. To date, sudden death due to exercise has not been reported in either overweight or hypertensive youth in the absence of other cardiovascular abnormalities, such as hypertrophic cardiomyopathy [18, 19]. Furthermore, studies of exercise stress testing in children and adolescents with hypertension, although limited in number, show no evidence of cardiovascular complications related to either dynamic or isometric exercise [14, 19].

The issue of sports participation in hypertensive children and adolescents has been considered by several expert panels, including the 36th Bethesda Conference, the NHBPEP Working Group, and the American Academy of Pediatrics Committee on Sports Medicine and Fitness. Competitive sports participation and highly static sports should be limited only in the presence of uncontrolled stage 2 hypertension or target-organ damage [2, 12, 20]. Cardiovascular conditioning less rigorous than competitive sports activity may not need to be restricted, and sports eligibility can resume once blood pressure is adequately controlled [12, 20]. Alpert has made the case for not limiting competitive sports activity in hypertensive youth, given the lack of evidence of any risk and given the importance, both psychologically and physically, of sports participation [19]. A generalized conditioning program should also be implemented for hypertensive children and adolescents participating in competitive sports [21].

Physicians are sometimes hesitant to allow hypertensive athletes to continue weight lifting. The concern comes from the extreme elevations in blood pressure (as high as 480/350 mmHg) and rare cases of subarachnoid hemorrhage reported in a small number of elite power lifters [22]. However, randomized, controlled trials in adults show that progressive resistance training can lower resting blood pressure, and no harmful effects of resistance training have been demonstrated in hypertensive children and adolescents [11, 16, 19]. The American College of Sports Medicine recommends that athletes with hypertension establish a training program that includes both dynamic and resistance exercise [11]. Similarly, the NHBPEP Working Group has recognized the potential benefit of both aerobic exercise and resistance training, with the notable exception of power lifting [2].

Therapeutic lifestyle modification is essential for any child or adolescent with hypertension and should include regular physical activity, a diet limited in sodium but rich in fresh fruits and vegetables, fiber, and low-fat dairy products, and the avoidance of excess weight gain [2]. Regular exercise is beneficial for those suffering both obesity and hypertension, and weight loss programs that incorporate regular exercise are more effective than those using nutritional management alone [21].

In addition to the implementation of therapeutic lifestyle changes, some athletes will require pharmacologic therapy for hypertension management. The choice of medication should take into account side-effect profiles of particular importance to athletes [16]. Furthermore, physicians and athletes should be aware that the use of diuretics and beta-blockers has been banned by college sport and Olympic governing bodies [20]. In general, diuretics should be avoided in athletes, because of the risk of dehydration, heat illness, and electrolyte abnormalities. Beta-blockers should be avoided, due to the potential for reduction in maximal exercise capacity [16]. Angiotensin-converting enzyme inhibitors, angiotensin-II receptor blockers, and calcium channel blockers have low side-effect profiles, do not affect exercise tolerance, and are listed as acceptable drug classes for use in hypertensive children by the Working Group [2, 16]. Angiotensin-converting enzyme inhibitors and angiotensin-II receptor blockers are contraindicated in pregnancy, due to the risk of fetal damage, which has been reported even after exposure during the first trimester [23, 24]. Female athletes of child-bearing age treated with these agents should, therefore, be counseled to use contraception if sexually active.

### Hypertension in chronic kidney disease

*A. is a 6-year-old boy with autosomal dominant polycystic kidney disease. He was diagnosed at birth, due to the detection of bilateral flank masses on his initial physical examination in the newborns’ nursery. Recently, he has been noted to have intermittently elevated blood pressures during his follow-up visits to your office, with some readings as high as 130/78 mmHg. He has stage II CKD, with an estimated glomerular filtration rate (GFR) calculated by the Schwartz formula of 65 ml/min per 1.73 m*^*2*^. *Current medications include sodium bicarbonate, albuterol for asthma, and multivitamins.*

## Scope of the problem

Children with CKD are frequently hypertensive. The 2006 annual report of the North American Pediatric Renal Trials and Collaborative Studies (NAPRTCS) showed that 39.3% of children enrolled in the chronic renal insufficiency registry were taking antihypertensive medications [25]. The prevalence of hypertension is especially prominent in children with CKD due to polycystic kidney disease (83%) and glomerulonephritis (71%) [26]. Most recently, a preliminary analysis of 227 children enrolled in the Chronic Kidney Disease in Children (CKiD) study, an ongoing prospective, multi-center observational study of North American children with CKD, showed that 56% were on antihypertensive therapy and that the prevalence of hypertension increased with decreasing GFR [27]. Furthermore, studies of ambulatory blood pressure monitoring in children with CKD demonstrate a high prevalence of nocturnal hypertension [28]. The high prevalence of hypertension in children with CKD has significant implications, given the potential impact on cardiovascular outcomes and progression of kidney disease.

## Hypertension and cardiovascular risk

Cardiovascular disease is the most common cause of death in adults with CKD [29]. The National Kidney Foundation (NKF) Task Force on Cardiovascular Disease and the NKF-Kidney Disease Outcomes Quality Initiative (K/DOQI) Work Group on Hypertension and Antihypertensive Agents in Chronic Kidney Disease identified management of hypertension as essential to the decreasing of cardiovascular mortality in adults with chronic kidney disease [29–31].

Recently, it has become apparent that children with CKD are also at increased cardiovascular risk. Analyses of the US Renal Data Systems database showed that children with end-stage renal disease (ESRD) have a 1,000 fold higher cardiac death rate than the age-matched general population, and cardiac death accounted for 38% of total deaths in children on dialysis [32, 33]. Moreover, studies show that cardiovascular abnormalities are common in children with CKD, even prior to the development of end-stage kidney disease. Left ventricular hypertrophy (LVH) and increased carotid intima media thickness (IMT), early surrogate markers for cardiomyopathy and atherosclerosis, respectively, are common in cross-sectional studies of children with stage 2 to stage 4 CKD [30, 34]. The role of hypertension in the development of LVH and increased carotid IMT has been extensively reviewed in a previous Educational Feature highlighting the cardiovascular complications of CKD in children [35]. While there have been no longitudinal studies of the effect of treatment of hypertension on cardiovascular outcomes in children with CKD, it is likely that appropriate treatment of hypertension would ameliorate, in part, the high risk of cardiovascular disease in individuals with CKD from a young age.

## Hypertension and progression of CKD

The NKF Work Group on Hypertension and Antihypertensive Agents in Chronic Kidney Disease defined the slowing of the progression of CKD as a principal goal of antihypertensive therapy in CKD [31]. The Modification of Diet and Renal Disease (MDRD) study demonstrated that adults with proteinuria had a slower rate of progression of CKD when target mean arterial blood pressure was 92 mmHg than when it was 107 mmHg. The effect of more vigorous blood pressure control was most pronounced in patients with proteinuria >3 g/day, and there was no apparent benefit in adults with <1 g/day proteinuria [36]. A recent analysis of the NAPRTCS chronic renal insufficiency database suggests that hypertension plays a role in the progression of CKD in children as well. In that analysis, Cox proportional hazards modeling identified systolic hypertension as an independent predictor of CKD progression [37].

While the above data are convincing, prospectively generated data are needed if we are to understand more clearly the role that hypertension plays in progression of CKD in children and adolescents. The previously mentioned CKiD study [27] includes prospective casual and ambulatory blood pressure measurement as well as assessment of cardiac structure and function by echocardiography, all in conjunction with precise measurement of GFR by iohexol clearance [27, 30]. This study should answer many questions related to the role of blood pressure in progression of CKD in children.

## Treatment of hypertension in CKD patients

Many studies of adults with CKD demonstrate that antihypertensive regimens that include angiotensin-converting enzyme (ACE) inhibitors or angiotensin receptor blockers (ARBs) are more effective than regimens based on other classes of drugs in slowing progression of CKD, particularly in patients with proteinuria [38]. Some of this benefit may be due to other effects of these agents, such as reduction of glomerular fibrosis. Consensus guidelines recommend that adults with CKD should have a goal blood pressure <130/80 mmHg and that most adults with CKD should receive an ACE inhibitor or ARB [1, 31]. Whether ACE inhibition and level of blood pressure control affect progression of CKD in children is being prospectively evaluated by the Effect of Strict Blood Pressure Control and ACE Inhibition on Progression of CRF in Pediatric Patients (ESCAPE) trial, a multi-center, prospective trial being conducted in Europe. Patients with GFRs between 11 ml/min per 1.73 m^2^ body surface area and 80 ml/min per 1.73 m^2^ body surface area are randomly allocated to conventional or intensified blood pressure management with ramipril, aiming for the 50th to 95th percentile or below the 50th percentile, respectively, for 24 h mean arterial pressure [39]. Long-term follow-up of the ESCAPE cohort should help determine the effects of ACE inhibition in children with CKD. Published experience with ARB therapy in children with CKD is more limited, although single-center data suggest that losartan is well tolerated and confers sustained antihypertensive and renoprotective effects [40].

Combination therapy with ACE inhibition and ARB may be more beneficial than either class of medication given alone, although previous studies have been limited by the use of submaximal dosing of both the combination ACE inhibitor–ARB and the comparison single drug [38, 41]. Experience with the use of combination therapy with ACE inhibition and ARB in children with CKD is limited. Small single-center reports suggest an additive antiproteinuric effect, but concern has been raised regarding an increased risk of significant hyperkalemia [42, 43]. Multi-center, prospective trials comparing ACE inhibition alone with combined therapy in children with CKD are needed to establish the safety and efficacy of combined therapy.

Because fluid retention is a common cause of hypertension in CKD, diuretics are required in many patients to achieve target blood pressures. Thiazide diuretics are effective in lowering blood pressure and decrease cardiovascular risk in adults. Since thiazides are less effective at low levels of GFR, loop diuretics are preferred for patients with a GFR <30 ml/min per 1.73 m^2^ [31].

Recently, the role of aldosterone antagonism has received significant attention in the treatment of hypertensive patients with CKD. Aldosterone receptor antagonists have been shown to have beneficial effects on cardiac fibrosis and may also further ameliorate proteinuria in CKD when used in combination with an ACE inhibitor or ARB [44]. To date, only one study of children treated with aldosterone blockade has been published: a small case series of patients with Alport’s syndrome in whom addition of spironolactone to an ACE inhibitor or ACE inhibitor + ARB resulted in a further reduction in proteinuria without significant adverse effects [45]. This strategy will clearly need further studies to establish its role in the treatment of hypertensive children with CKD.

Studies of adults with CKD show that both dihydropyridine calcium channel blockers (CCBs; i.e., amlodipine, nifedipine, felodipine) and non-dihydropyridine CCBs (diltiazem, verapamil) are effective in lowering blood pressure. Non-dihydropyridine CCBs have the additional advantage of decreasing proteinuria and preserving renal function, whereas dihydropyridine CCBs do not have this effect, possibly because of a greater loss of renal autoregulation with dihydropyridines [46]. When used in combination with ACE inhibition, dihydropyridine CCBs do not detract from the renoprotective effects of renin–angiotensin blockade, but some authors recommend the avoidance of these agents as monotherapy in proteinuric renal disease [46]. Because there is little experience with non-dihydropyridine CCBs in children, the role of these potentially renoprotective agents will need to be determined in prospective, controlled trials before their routine use in children with CKD can be advocated.

The NHBPEP Working Group recommended that children with chronic renal disease have a target blood pressure <90th percentile for age, gender, and height and that ACE inhibitors or ARBs should be used preferentially in children with proteinuric renal disease [2]. A recent survey of North American pediatric nephrologists confirmed that the vast majority do use ACE inhibitors for first-line therapy of hypertension in children with proteinuric kidney disease [47]. However, preliminary analysis of the CKiD cohort study found that many children with elevated blood pressure, defined as blood pressure >90th percentile, were not on antihypertensive therapy, despite the NHBPEP Working Group recommendations [27]. Future studies of antihypertensive regimens in children with CKD will help guide best practice and, hopefully, improve long-term outcomes.

### The obese adolescent with metabolic syndrome

*J. is a 17-year-old girl referred by the obesity clinic for evaluation of elevated blood pressures. In addition to blood pressure readings of 135–147/70–82 mmHg, physical examination findings include height >95th percentile, body mass index (BMI) 47 kg/m*^*2*^, *acanthosis nigricans and striae on the proximal extremities and abdomen. Her regular exercise is limited to gymnastics class twice per week (30 min per session), and her diet includes many fast-food meals and at least four servings of soda daily. Her mother is also obese and has type 2 diabetes; her paternal grandfather died at age 50 years from a myocardial infarction. Laboratory evaluation reveals a fasting glucose concentration of 110 mg/dl, fasting insulin 50 μU/ml, total cholesterol 185 mg/dl, high-density lipoprotein (HDL) cholesterol 32 mg/dl, low-density lipoprotein (LDL) cholesterol 153 mg/dl and triglycerides 262 mg/dl.*

Obesity has reached epidemic proportions among children and adolescents in the USA [48], and its prevalence appears to be increasing in other countries as well [49]. It is, therefore, not surprising that complications of obesity, such as elevated blood pressure, are becoming more common. In the Houston public schools, for example, a recent screening study demonstrated that up to 5% of students had elevated blood pressure [50], which was much greater than the traditional estimated prevalence of 1–2% [51]. Persistently elevated blood pressure was present in up to 11% of those whose BMI was ≥95th percentile for age and gender.

While the pathogenesis of hypertension in the obese is complex and not fully understood, two hormonal abnormalities are likely involved: hyperinsulinemia, a consequence of peripheral insulin resistance (primarily in the skeletal muscle), and hyperleptinemia, a consequence of the increased mass of adipose tissue. Hyperinsulinemia contributes to the development of hypertension by at least three proposed mechanisms: altered renal sodium handling, increased vascular resistance, and increased activity of the sympathetic nervous system (SNS) [52, 53]. Further activation of the SNS is caused by the increased levels of leptin, circulating levels of which have been correlated to blood pressure [54]. Activation of the SNS has been implicated in clinical studies of obese children and may explain the predominance of isolated systolic hypertension in this group [55].

Hypertension is a defining characteristic of the metabolic syndrome (MS), a constellation of abnormal clinical and laboratory findings, including central obesity, insulin resistance and dyslipidemia. The MS is associated with the development of atherosclerosis and is a strong independent predictor of cardiovascular events in hypertensive adults [56]. At present, there is no consensus on how to define the MS in children and adolescents, nor is there consensus on whether the MS in the young should be viewed primarily as a precursor to type 2 diabetes, or as a predictor of future cardiovascular disease [57]. At least one group of investigators has recently demonstrated that components of the MS are strongly associated with left ventricular hypertrophy and other target-organ effects of hypertension [58], which would be consistent with data from studies of adults. Given this, the threshold for initiating treatment with antihypertensive medications should probably be lower in children and adolescents with the MS than in non-obese patients with primary hypertension.

Before the patient proceeds to medications, however, lifestyle modifications should be implemented. Although success may be difficult to achieve, the benefits have been well documented in the literature. Two recent studies have demonstrated that short-term diet and exercise can result in weight loss, reduced blood pressure, and improvements in the abnormal laboratory findings associated with the MS in overweight children and adolescents [59, 60]. Furthermore, exercise was proven to be more beneficial than metformin in preventing the development of frank type 2 diabetes in high risk adults [61]. These studies support the recommendation made by the NHBPEP Working Group that therapeutic lifestyle changes should be considered primary therapy for obesity-related hypertension in children and adolescents.

Pharmacologic management of hypertension in the MS has two unique aspects to consider: first, the cardiovascular effects of insulin-sensitizing medications, and second, the effects of some classes of antihypertensive agents on insulin sensitivity [62]. Metformin, thiazolidinedione peroxisome proliferator-activated receptor-γ (PPAR-γ) agonists and the glucose oxidase inhibitor acarbose have all been shown to have modest blood pressure lowering effects in clinical trials in adults, but this effect should be considered adjunctive to other antihypertensive therapies.

The tendency of diuretics and beta-adrenergic blockers to affect glucose metabolism is well-known and needs to be considered when the physician is choosing an antihypertensive agent for obese children with signs of the MS [62]. The combination of these two drug classes is felt to have significant diabetogenic potential [63] and should probably be avoided as initial therapy in this patient population. This stands in contrast to non-obese patients with primary hypertension, in whom diuretics and beta-adrenergic blockers would be appropriate as first-line therapy, unless a specific contraindication, such as severe asthma, was present. These drug classes do have a role in patients with the MS, however, usually as second- or third-line agents when drugs from other classes are not sufficient to achieve target blood pressure [4]. On the other hand, CCBs, ACE inhibitors, ARBs and alpha-adrenergic blockers have no detrimental effects on glucose metabolism and are acceptable for use as initial agents in obese patients with the MS—just as they would in non-obese hypertensive patients.

The hypertensive child or adolescent with the MS also illustrates another important point in the therapy of hypertension, namely the need for other cardiovascular risk factors to monitor on an ongoing basis during treatment. Screening for dyslipidemia and impaired glucose tolerance should be performed on a regular basis, not only during the initial evaluation as recommended by the NHBPEP Working Group [2], but probably at least annually or semi-annually. Similar screening should also be included in the treatment of non-obese patients with primary hypertension, although perhaps not as often as for obese patients with the MS. While early exposure to the multiple cardiovascular risk factors present in the MS clearly increases the risk of early development of atherosclerosis [64], the role of imaging in screening for early signs of atherosclerosis in the young remains to be determined [65]. However, given the increasing evidence that the MS is a precursor of adult cardiovascular disease [57], vigilance and early intervention are essential in order to optimize long-term outcomes for these patients.

## Conclusions

The most recent NHBPEP Working Group guidelines, published in 2004, provide comprehensive recommendations regarding the evaluation, diagnosis, and therapy of children and adolescents with elevated blood pressure [2]. Persistently elevated blood pressures are required for the diagnosis of hypertension, so that the physician may avoid an incorrect diagnosis in a child with white-coat hypertension. Ambulatory blood pressure monitoring is more reliable than clinic blood pressure measurements in the evaluation for white-coat hypertension; therefore, confirmation of hypertension by ambulatory monitoring should be strongly considered prior to the initiation of pharmacologic therapy. Non-pharmacologic therapeutic life-style modifications should be instituted in all hypertensive patients. When indicated, antihypertensive medication should generally begin with a single agent appropriate to the underlying pathophysiology of the patient’s hypertension [4]. Recent legislative initiatives have resulted in a significant increase in antihypertensive medications approved for use in children. However, there have been no comparative studies of efficacy of antihypertensive medications in childhood hypertension to guide pharmacologic therapy. The diagnostic evaluation and therapeutic approach to the child or adolescent with hypertension should take into account the particular clinical situation of the patient, as illustrated in the case discussions.

## Questions (answers appear after References)

1. Recently issued recommendations for staging hypertension in children and adolescents provide guidance for clinicians in evaluating patients with elevated blood pressure. The new staging system recommends immediate institution of antihypertensive medications for patients with BP in which of the following stages:
  - Stage 2 hypertension
  - Normal blood pressure
  - Stage 1 hypertension
  - Pre-hypertension
2. Diuretics should always be used in the treatment of athletes with hypertension because of the frequency of high-salt diets in young athletes.
  - True
  - False
3. Renal insufficiency and proteinuria are common in children with CKD, even those with non-glomerular diseases. When these are present, which of the following drug classes should be considered as appropriate therapy?
  - Dihydropyridine calcium channel blockers
  - Angiotensin receptor antagonists
  - Centrally acting agents
  - Beta-adrenergic blockers
4. The NHBPEP Working Group recommends that children with renal disease have a target blood pressure <90th percentile for age, gender, and height and that ACE inhibitors or ARBs be used preferentially for children with proteinuric renal disease.
  - True
  - False
5. A 16-year-old obese boy with hypertension and type 2 diabetes has been referred to you for evaluation. His office BP is 145/82 mmHg. An echocardiogram indicates that has LVH, and he has a mildly elevated urinary microalbumin to creatinine ratio. The referring physician prescribed hydrochlorothiazide 50 mg daily. Your recommendation should be:
  - Order ambulatory BP monitoring to assess his blood pressure control
  - Add metoprolol 100 mg daily to reduce his blood pressure further
  - Start him on regular aerobic exercise and a low-sodium diet
  - Stop the hydrochlorothiazide and start lisinopril 10 mg daily
